# The complete mitochondrial genomes of five lichenized fungi in the genus *Usnea* (Ascomycota: Parmeliaceae)

**DOI:** 10.1080/23802359.2018.1445485

**Published:** 2018-02-28

**Authors:** Erik R. Funk, Alexander N. Adams, Sarah M. Spotten, Roxanne A. Van Hove, Kristina T. Whittington, Kyle G. Keepers, Cloe S. Pogoda, James C. Lendemer, Erin A. Tripp, Nolan C. Kane

**Affiliations:** a Department of Ecology and Evolutionary Biology, University of Colorado, Boulder, CO, USA;; b Department of Molecular, Cellular and Developmental Biology, University of Colorado, Boulder, CO, USA;; c Institute of Systematic Botany, The New York Botanical Garden, Bronx, NY, USA;; d Museum of Natural History, University of Colorado, Boulder, CO, USA

**Keywords:** Symbiosis, *Usnea halei*, *Usnea mutabilis*, *Usnea subfusca*, *Usnea subgracilis*, *Usnea subscabrosa*

## Abstract

Known colloquially as ‘Old Man’s Beard’, *Usnea* is a genus of lichenized Ascomycete fungi characterized by having a fruticose growth form and cartilaginous central axis. The complete mitochondrial genomes of *Usnea halei*, *U. mutabilis*, *U. subfusca*, *U. subgracilis*, and *U. subscabrosa* were sequenced using Illumina data and then assembled *de novo*. These mitogenomes ranged in size from 52,486 bp (*U. subfusca*) to 94,464 bp (*U. subgracilis*). All were characterized by having high levels of intronic and intergenic variation, such as ORFs that encode proteins with homology to two homing endonuclease types, LAGLIDADG and GIY-YIG. Genes annotated within these mitogenomes include 14 protein-coding genes, the large and small ribosomal subunits (LSU and SSU), and 23–26 tRNAs. Notably, the *atp9* gene was absent from each genome. Genomic synteny was highly conserved across the five species. Five conserved mitochondrial genes (*nad2*, *nad4*, *cox1*, *cox2*, and *cox3*) were used to infer a best estimate maximum likelihood phylogeny among these five *Usnea* and other relatives, which yielded relationships consistent with prior published phylogenies.

## Introduction

*Usnea* is a genus of lichenized Ascomycete fungi that represents one of the most species-rich genera in Parmeliaceae, which is a family of more than 2000 species and 77 genera (Crespo et al. 2007; Lücking et al. 2016). *Usnea* has a cosmopolitan distribution, being found on every continent (Halonen 2000), and contains both saxicolous (rock-dwelling) and corticolous (bark-dwelling) species (Clerc and Herrera-Campos 1997; Clerc 1998; Halonen 2000). The genus on the whole is characterized by having a fruticose growth form and a cartilaginous central axis (Clerc 1998). Known colloquially as ‘Old Man’s Beard’, species of *Usnea* are charismatic and highly recognizable lichens across the globe (Brodo et al. 2001). Species are characteristically yellowish-green in colour due to the production of usnic acid, which is thought to have a multitude of potential functions including having antibiotic, antimycotic, and antiprotozoal properties (Lauterwein et al. 1995; Fournet et al. 1997; Giordano et al. 1997; Cocchietto et al. 2002). Here, we present newly sequenced and annotated mitochondrial genomes from five species of *Usnea* that occur in the southern Appalachian lichen biodiversity hotspot, located in eastern North America (Lendemer et al. 2013; Tripp and Lendemer in press; Tripp et al. in review). Two of these species (*U. halei* and *U. subfusca*) are endemic to eastern North America and have centres of distributions in the region (Clerc and Herrera-Campos 1997; Lendemer et al. 2013).

## Methods

Samples of *Usnea halei* (voucher Lendemer 46374; Lat/Long: 35.1356,–83.1911; NCBI accession MG722979), *U. mutabilis* (voucher Lendemer 49260; Lat/long: 34.3679,–85.6299; NCBI accession MG920803), *U. subfusca* (voucher Lendemer 46309; Lat/Long: 35.61,–83.4467; NCBI accession MG720812), *U. subgracilis* (voucher Lendemer 48717; Lat/Long: 35.1036,–83.2075; NCBI accession MG720066), and *U. subscabrosa* (voucher Lendemer 46747; Lat/long: 35.4372,–83.7481; NCBI accession MG720452) were collected by J.C. Lendemer, E.A. Tripp, K.G. Keepers and K.H. White from the Southern Appalachian Mountains in the United States between 2015 and 2017. Genomic DNA was extracted using a Qiagen DNeasy 96 plant kit, with the protocol modified to include a 10 min 65 °C incubation step for ground material in lysis buffer and a 100% ethanol wash before final drying of the membrane prior to elution. Genomic libraries were prepared using Nextera^®^ XT DNA library prep kits (Illumina^®^) and each sample was barcoded using the unique dual index adapters Nextera^®^ i5 and i7. Samples that passed QC were processed for paired-end 150 base pair read sequencing on the Illumina NextSeq^®^ at University of Colorado’s BioFrontiers Institute Next-Generation Sequencing Facility in Boulder, Colorado. Genomic reads were trimmed using Trimmomatic-0.36 with the parameters ‘ILLUMINACLIP:NexteraPE-PE.fa:2:20:10 MINLEN:140 LEADING:20 TRAILING:20’ (Bolger et al. 2014). Trimmed reads were assembled *de novo* using SPAdes v.3.9, with the parameters ‘-meta -k 35,55,85’ (Bankevich et al. 2012). Fungal mitochondrial contigs were identified using a command-line BLAST against a set of fungal mitochondrial proteins derived from the lichenized fungus *Peltigera dolichorrhiza* (NCBI accession KT946595), which were then circularized and error-corrected. Annotations of genomic features were initiated using DOGMA (Wyman et al. 2004) and completed in NCBI’s Sequin 15.10 (Bethesda, MD). Genome assemblies and annotations were performed by undergraduate and graduate students enrolled in N. Kane's Genomics class at the University of Colorado, Boulder.

## Phylogenetic analysis

A maximum likelihood phylogenetic hypothesis was constructed using sequence data from five loci (*cox3*, *nad2*, *nad4*, *cox1*, and *cox2*), which were concatenated prior to analysis. Outgroup sequences were obtained from NCBI: *O. vulgata* [KY315997], *P. ostiolata* [KY346830], *G. americanus* [NC_034790], *H. speciosa* [KY328643], *L. hirsutum* [NC_034928], *P. corallina* [NC_034779], *C. rangiferina* [KY460674], *C. uncialis* [KY352404], *P. stuppeum* [KY362439], *I. aleurites* [KY352227], *H. vittata* [KY362374], *M. subsimilis* [KY352491], *A. fallacina* [MG711470], *U. cornuta* [KY100278], *U. ceratina* [KX987159], and *U. pensylvanica* [KY321923]. Sequences were aligned in MEGA v.7.0.26 (Kumar et al. 2016) using the MUSCLE aligner (Edgar 2004). The resulting alignment was manually manipulated to exclude regions of the alignment that contained excessive missing data (available at Dryad Zenodo, record 1185346). Gaps were treated as missing data. The ML tree was inferred using RAxML v.8.2.10 (Stamatakis 2014). To assess support among relationships, we conducted 250 bootstrap replicates using the autoMRE criterion in RAxML and from this distribution, a consensus topology was generated. Bootstrap support values were mapped onto the most likely tree deriving from the above search. The tree was rooted using *Opegrapha vulgata*.

## Results and discussion

### Mitochondrial genome content and organization

The lengths of *Usnea* mitochondrial genomes in this study varied from 52,486 bp (*U. subfusca*) to 94,464 bp (*U. subgracilis*). The mitochondrial genomes of the other three species were of intermediate lengths: 61,314 bp (*U. mutabilis*), 78,464 bp (*U. subscabrosa*), and 82,851 bp (*U. halei*). The genomes presented here contained a conserved set of 14 protein coding genes (*cob*, *cox1*, *cox2*, *cox3*, *nad1*, *nad2*, *nad3*, *nad4*, *nad4L*, *nad5*, *nad6*, *atp6*, *atp8*, and *rps3*). Notably, all lacked a key mitochondrial gene (*atp9*), which has previously been shown to have been lost evolutionarily from three different lineages of lichens, one of which includes Parmeliaceae (Pogoda et al. 2018). Synteny of protein-coding genes in the mitogenomes was highly conserved across the five species of *Usnea*, with no differences in overall gene order and no inversions detected.

### Introns and intergenic spacer regions

Among the five samples, non-coding DNA contributed to overall genome size: *U. subfusca* has the smallest mitochondrial genome and also the least amount of intronic sequence. The three largest genomes (*U. subscabrosa*, *U. halei*, and *U. subgracilis*) contained variable lengths of intronic sequences, but all had approximately two to four times more total intergenic sequence compared to the other genomes. Notably, the three largest genomes also contained a large intergenic region between *atp8* and *cob*, which contained multiple fragments of a DNA polymerase (*dpo*) and ranged in size from approximately 4 kb in *U. subgracilis* to 17.9 kb in *U. subscabrosa*. All five genomes contained a large intergenic gap between the LSU and *nad2* genes, ranging in size from 4.8 to 7.7 kb and containing 7–13 tRNA sequences. *Usnea subscabrosa* also contained an ORF with homology to a GIY-YIG homing endonuclease in this region.

### Open reading frames

*Usnea mutabilis* had multiple ORFs throughout its mitogenome. The longest ORF (1193 bp) was found preceding *nad5* and is likely co-transcribed with a 3′ ORF that shares the stop codon of the protein coding gene. Two hypothetical proteins were located between *nad4* and *rps3* and between *cox1* and *nad4*, having lengths of 311 bp and 758 bp, respectively. We found that *U. mutabilis* possessed three ORFs with homology to homing endonucleases located in the introns of the *cox1* and *LSU* genes. *Usnea subfusca* was found to have two ORFs, one with homology to a GIY-YIG homing endonuclease that was 1445 bp and was located at the 3′ end of the *nad1* gene.

### Phylogenetic history

**Figure 1.**
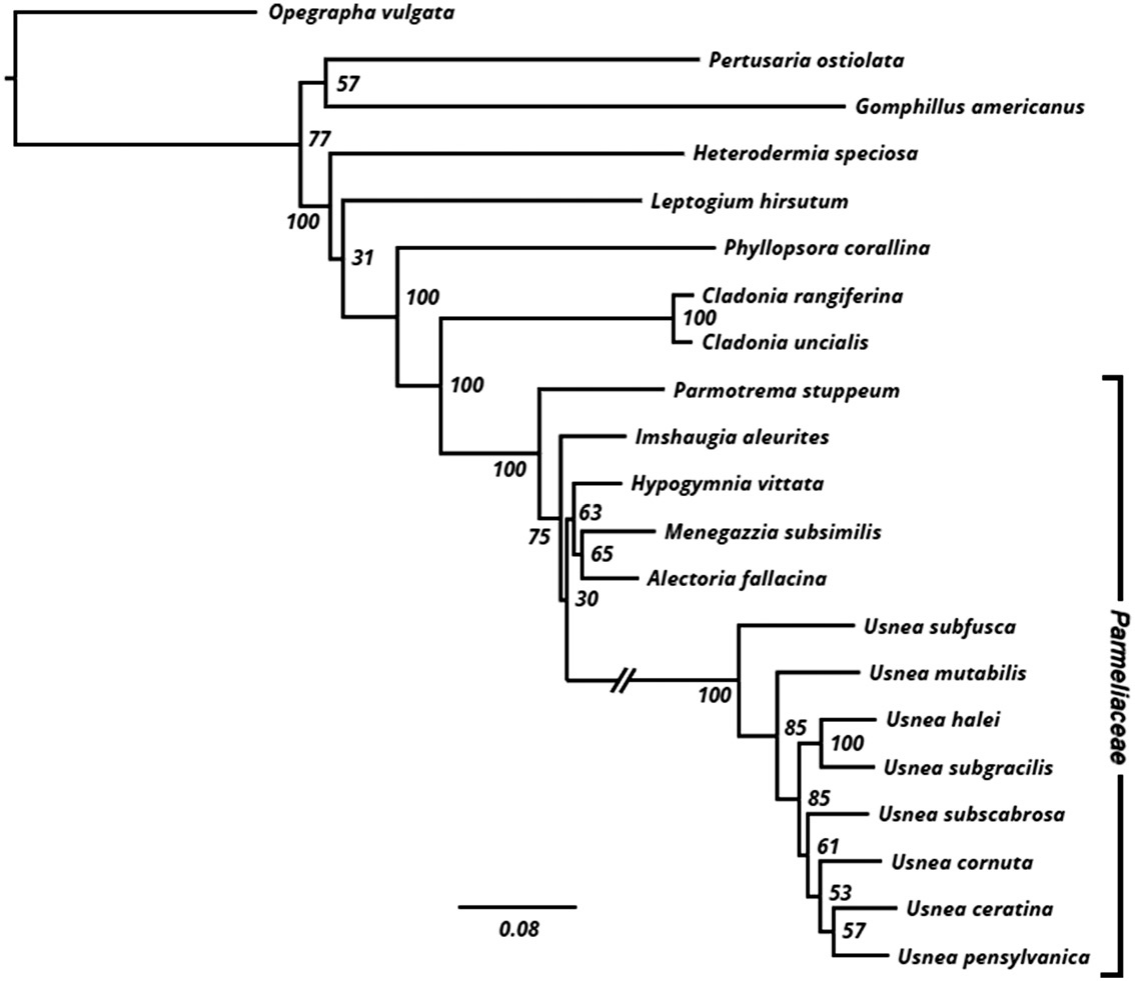
Phylogenetic relationships among *Usnea* and other lichens inferred using maximum likelihood (see Methods section). Numbers next to nodes represent bootstrap support mapped onto the most likely tree (–ln *L* = 68803.11). The long branch leading to *Usnea* was shortened (hashes) for the figure.

All eight species of *Usnea* (the five presented here, along with three previously sequenced species obtained from NCBI) were recovered as monophyletic with 100% bootstrap support (Figure 1). Monophyly of Parmeliaceae as a whole was also well supported (BS = 100%), with *Imshaugia*, *Hypogymnia*, *Menegazzia*, and *Alectoria* recovered as sister to *Usnea*. This topology is in general agreement with previously published phylogenies (e.g. Crespo et al. 2010; Miadlikowska et al. 2014).

## Conclusions

The five newly sequenced, assembled, and annotated *Usnea* mitogenomes exhibited a large range in size, with intronic and intergenic regions of variable lengths associated with changes in genome size. These mitochondrial genomes coded for a variable set of ORFs, but all contained the same set of 14 protein-coding genes. Results from our study confirm earlier findings (Pogoda et al. 2018) that members of Parmeliaceae have lost a key gene involved in energy production, *atp9*, lending genomic evidence of the obligate nature of this symbiotic relationship. We thus infer that these five species of *Usnea* are likely dependent on their photosynthetic partner(s) for energy production. Finally, these data provide an important resource for further study of the evolution of parasitic, selfish elements such as LAGLIDADG and GIY-YIG type homing endonucleases in *Usnea* and related lichens. The present study yields new resources for further study of lichen mitochondrial genome evolution and co-evolution with obligate symbiotic partners.
